# Relationship Between Renal Resistive Index and Retinal Vascular Density in Individuals with Hypertension

**DOI:** 10.3390/biomedicines13020312

**Published:** 2025-01-28

**Authors:** Caterina Carollo, Maria Vadalà, Alessandra Sorce, Nicola Sinatra, Emanuele Orlando, Emanuele Cirafici, Miriam Bennici, Riccardo Polosa, Vincenza Maria Elena Bonfiglio, Giuseppe Mulè, Giulio Geraci

**Affiliations:** 1 Unit of Nephrology and Dialysis, Hypertension Excellence Centre, Department of Health Promotion, Mother and Child Care, Internal Medicine and Medical Specialties (PROMISE), University of Palermo, 90133 Palermo, Italyemanuele.cirafici@community.unipa.it (E.C.); giuseppe.mule@unipa.it (G.M.); 2 Biomedicine, Neuroscience and Advance Diagnostic (BIND) Department, University of Palermo, 90133 Palermo, Italy; maria.vadala@unipa.it (M.V.);; 3 UOSD Nefrologia e Dialisi, Ospedale Paolo Borsellino, 91025 Marsala, Italy; sinatra.nicola@libero.it; 4 Department of Health Promotion, Mother and Child Care, Internal Medicine and Medical Specialties (PROMISE), University of Palermo, 90133 Palermo, Italy; emanuele.orlando01@unipa.it; 5 Department of Medicine and Surgery, “Kore” University of Enna, 94100 Enna, Italygiulio.geraci@unikore.it (G.G.)

**Keywords:** hypertension, microcirculation, angio-OCT, renal resistive index

## Abstract

**Background/Objectives**: Considering the physiological analogies between the eye and the kidney, this study aimed to investigate the potential relationship between retinal vascular density, assessed using Optical Coherence Tomography Angiography (OCT-A), and the renal resistive index (RRI) in patients with arterial hypertension. **Methods**: A total of 82 hypertensive patients (mean age 48 ± 13) were enrolled in the study. Participants underwent routine biochemical evaluations, office-based blood pressure measurement, 24 h ambulatory blood pressure monitoring, OCT-A imaging, and renal Doppler ultrasound examinations. **Results**: The mean RRI in the study population was 0.616 ± 0.06. Participants were divided into two groups based on the 75th percentile threshold of the RRI distribution (0.66, 95% CI 0.64–0.68). The group with RRI > 75th percentile, which appeared to have a higher number of smokers, exhibited significantly higher mean triglyceride and urinary albumin excretion (UAE) levels and a significantly reduced estimated glomerular filtration rate (eGFR) as compared to the group with RRI < 75th percentile. Among the hemodynamic parameters, 24 h pulse pressure (PP), daytime and nighttime PP, and nighttime systolic blood pressure (SBP) were significantly higher in the group with RRI > 75th percentile. Regarding retinal vascular density indices, the only significant difference was observed in the deep foveal vascular plexus, which displayed a reduced density in the group with RRI > 75th percentile. Logistic regression analysis revealed that RRI > 75th percentile was independently associated with increased nighttime mean pulse pressure (OR = 1.13, 95% CI: 1.049–1.221, *p* = 0.0014) and reduced deep foveal vascular density (OR = −0.5026, 95% CI: 1.0493–1.2211, *p* = 0.0044). **Conclusions**: Our findings demonstrate that ocular microvascular alterations are associated with RRI, a marker with a well-established prognostic value for renal disease progression and systemic macrovascular dysfunction. These results further substantiate the close relationship between renal and ocular microcirculation.

## 1. Introduction

Systemic hypertension significantly impacts both the structure and function of the microvascular system [1]. The development of microvascular damage, particularly microvascular rarefaction, is believed to be a crucial pathological feature of hypertension.

Changes in the structure and function of the microvasculature, in addition to being linked to the development of hypertension by influencing flow resistance and tissue perfusion, underlie much of the organ damage associated with arterial hypertension and appear to be crucial to its pathogenesis and progression [2,3].

Since the eye and kidney share common developmental, structural, and pathogenic pathways, changes in eye microcirculation could therefore be correlated with intrarenal hemodynamic damage, which has been associated with endothelial dysfunction, subclinical organ damage, and adverse cardiovascular outcomes, and appears to be a good indicator of systemic morphofunctional arterial impairment, particularly in hypertensive individuals with or without normal renal function [2,4,5,6,7].

The ocular microcirculatory system is readily accessible for clinical and morphological assessment, allowing for repeated and non-invasive examination [8,9]. This provides a unique opportunity to observe the vascular network when impacted by systemic conditions like hypertension, diabetes mellitus, and chronic kidney disease (CKD). The integration of optical coherence tomography angiography (OCTA) into clinical practice has introduced a dependable method for examining retinal and choroidal circulation from a morphological perspective.

There is a substantial body of evidence that the arterial stiffness predicts future cardiovascular and total mortality risk in various patient populations, and clinical studies have associated its elevation with the development of macrovascular and microvascular damage [10,11,12].

The renal resistive index (RRI) is a sonographic measure of the intrarenal arteries, calculated as (peak systolic velocity—end-diastolic velocity)/peak systolic velocity. RRI measurement is relatively simple, and Doppler ultrasonography is a non-invasive, cost-effective, and rapid imaging technique that provides real-time visualization of blood flow within the renal vessels.

The RRI is a well-established prognostic marker for both renal disease progression and systemic macrovascular alterations [13,14,15]. Despite the evidence of strong prognostic potential, there is no universal optimal cut-off value for the RRI. Most of the dedicated studies report a range between 0.5 and 0.7 [16,17].

Since both renal and ocular microcirculation are influenced by similar mechanisms of hemodynamic and endothelial dysfunction, we aimed to investigate whether changes in the renal resistive index could be correlated with alterations in ocular microcirculation.

Such a relationship could provide valuable insights into the systemic nature of vascular diseases, where both the kidney and the eye may reflect shared pathophysiological mechanisms, offering potential for the early diagnosis and monitoring of cardiovascular and renal conditions.

## 2. Materials and Methods

The population for this study was selected from hypertensive patients attending the ESH Hypertension Excellence Centre Outpatient clinic of our Nephrology and Hypertension Unit. Enrolment was conducted in accordance with the following exclusion criteria:

- Age < 20 years or >70 years;
- Known diabetes or fasting glucose levels > 126 mg/dL;
- Pregnancy;
- Systemic or ocular diseases (e.g., glaucoma, uveitis, high myopia, macular degeneration) or a history of ocular surgeries potentially causing retinal or choroidal damage;
- Nephroparenchymal, renovascular, malignant, or endocrine hypertension, or obstructive sleep apnea syndrome;
- Hereditary or non-hereditary kidney diseases, nephritic syndrome, or overt proteinuria/hematuria;
- Estimated glomerular filtration rate (eGFR) < 15 mL/min/1.73 m^2^ or renal replacement therapy (transplant or dialysis);
- Rapid decline in renal function, defined as a >25% reduction in eGFR or a >1.5-fold increase in serum creatinine levels from baseline [18];
- Poor-quality ultrasound imaging or abnormal renal morphology, as previously described [6];
- History or clinical evidence of heart failure (NYHA class II–IV), coronary artery disease, or cerebrovascular disease;
- Major non-cardiovascular conditions (e.g., liver cirrhosis, chronic obstructive pulmonary disease, or a history of malignancies);
- Conditions interfering with reliable blood pressure (BP) measurements using the oscillometric technique, such as atrial fibrillation, frequent ectopic beats, or second/third-degree atrioventricular blocks.

Patients with an arm circumference exceeding 32 cm were not excluded. Instead, appropriately sized cuffs were used to ensure accurate BP measurements.

The study protocol adhered to the principles of the Declaration of Helsinki, and written informed consent was obtained from all participants.

### 2.1. Study Design

A total of 82 patients with arterial hypertension (mean age 48.78 ± 12.61 years; 79% male) were enrolled and underwent the following assessments:

- Routine biochemical evaluations;
- 24 h ambulatory brachial blood pressure monitoring (ABPM) using an oscillometric BP Lab Vasotens device;
- Optical Coherence Tomography Angiography (OCT-A);
- Renal Doppler ultrasound.

#### 2.1.1. Blood Pressure Measurement

Office-based blood pressure (BP) was determined as the average of three consecutive measurements taken at two-minute intervals using an electronic oscillometric device (WatchBP Office, Microlife AG, Widnau, Switzerland) after five minutes of seated rest. For the 24 h ambulatory blood pressure monitoring (ABPM), an oscillometric device (BP Lab Vasotens) was used, adhering to current European Society of Hypertension (ESH) guidelines for proper recording [19]. Measurements were automatically taken at 15 min intervals during the daytime and at 20 min intervals during nighttime. The cuff was placed around the non-dominant arm, and patients were instructed to keep the arm still and avoid any movement during the readings. Oscillations within the cuff were recorded during gradual deflation to measure the BP.

The 24 h pulse pressure (24 h PP) is defined as the difference between 24 h systolic blood pressure (SBP) and 24 h diastolic blood pressure (DBP). Daytime and nighttime pulse pressure are calculated using the same method within their respective time periods.

#### 2.1.2. Biochemical Parameters

Routine biochemical parameters were determined using the standard techniques with an automated analyzer (Boehringer Mannheim for Hitachi system 911, Mannheim, Germany). Glomerular filtration rate (GFR) was estimated using the CKD-EPI equation.

#### 2.1.3. Ophthalmological Evaluation

A comprehensive ophthalmological examination was performed on all the patients, including corrected visual acuity measurement using the Early Treatment Diabetic Retinopathy Study (ETDRS) charts [20]. Intraocular pressure (IOP) was assessed with a Goldmann applanation tonometer. Anterior and posterior segment evaluations were conducted using a slit lamp under pharmacologically induced mydriasis with 1% phenylephrine drops. Retinal imaging was performed using a swept-source optical coherence tomography (SS-OCT) device (Triton; Topcon Inc., Itabashi, Japan).

All scans were conducted by a single operator between 10:00 A.M. and 12:00 P.M. The right eye was examined first, followed by a standardized scanning protocol. Poor-quality scans were repeated or discarded. Since no significant differences were observed between the two eyes, only one eye per subject was selected for analysis using a random number generator. If the selected eye’s scan quality was deemed insufficient, the contralateral eye was analyzed.

#### 2.1.4. Retinal Imaging Protocols

The following OCT scan protocols were used for each eye:

- 3D 7 × 7H Scan;
- Macular Radial 6.0 Scan;
- Angio-OCT 4.5 Scan,

Retinal thickness (from the internal limiting membrane to the inner surface of the retinal pigment epithelium) and choroidal thickness (from the outer surface of the retinal pigment epithelium to the sclera) were automatically calculated using the OCT mapping software. Measurements were presented as mean ± standard deviation across the nine regions defined by the ETDRS study grid.

The ETDRS grid divides the macula and choroid into nine fields. The grid, centered on the fovea, consists of three concentric rings with diameters of 1 mm, 3 mm, and 6 mm. The innermost and outermost rings are further divided into temporal, nasal, inferior, and superior quadrants, enabling detailed topographic analysis.

#### 2.1.5. Quantitative Analysis

OCT angiograms centered on the fovea (4.5 × 4.5 mm; 320 × 320 pixels) were analyzed to evaluate the superficial vascular plexus and the deep vascular plexus. The superficial vascular plexus is located within the ganglion cell layer, while the intermediate and deep vascular plexuses are positioned above and below the inner nuclear layer, collectively referred to as the deep capillary complex. The perimeter of the foveal avascular zone (FAZ) was manually delineated by a single operator on all the images of the superficial plexus. Using the OCT software (DRI Triton version 1.04E—1.36.2, Topcon Inc., Tokyo, Japan), the FAZ area was automatically calculated.

To minimize the statistical errors associated with subjective measurements, the final data used in the study were derived from the average of two independent measurements.

Image processing and measurements of retinal vascular density were performed using Image J software, version 1.49 (National Institutes of Health, Bethesda, MD, USA). Retinal vascular network images were generated using an automatic thresholding algorithm. Vascular density was defined as the percentage of the area occupied by blood vessels, with vessels identified as pixels exceeding the defined threshold value.

The calculations were performed on the following two regions of interest (ROIs): the foveal and parafoveal regions. The foveal ROI was defined as a central circle with a diameter of 120 pixels (1.2 mm), while the parafoveal ROI was defined as an annulus 91 pixels wide surrounding the foveal region.

#### 2.1.6. Ultrasound Evaluation

Intrarenal duplex ultrasonography was performed on all patients by a single trained operator blinded to the clinical data. The measurements were obtained using a GE Logiq P5-PRO device with a 4 MHz transducer and a Doppler frequency of 2.5 MHz. Patients were in a supine position, and the Doppler signal was obtained from the interlobar arteries by positioning the sample volume at the cortico-medullary junction.

The Renal Resistive Index (RRI) was calculated using the following formula:

RRI = Peak Systolic Velocity − End-Diastolic Velocity/Peak Systolic Velocity

Values were averaged from six measurements (three per kidney) after conducting hypothesis testing and finding no statistically significant difference in the RRI values between the two kidneys. Doppler angles were maintained at <60°, ensuring no renal compression or Valsalva maneuver, which could artificially elevate RRI.

#### 2.1.7. Renal Function Parameters

In patients with urinalysis showing proteinuria, even in trace amounts, or microalbuminuria detected via semiquantitative dipstick evaluation, a 24 h urinary albumin excretion assay was requested.

Albuminuria was measured using a turbidimetric method and expressed in mg/day. Serum creatinine levels were determined using a standardized enzymatic method (Creatinine Plus, Roche Diagnostics). The glomerular filtration rate (GFR) was estimated using the CKD-EPI (Chronic Kidney Disease Epidemiology Collaboration) equation.

The study population was stratified into two groups based on the intrarenal parenchymal renal resistive index (RRI) values above and below the 75th percentile of the RRI distribution; the threshold value was set at 0.66.

### 2.2. Statistical Analysis

Statistical analysis was conducted using Medcalc version 15 and IBM-SPSS version 26 software packages. The distribution of continuous variables was evaluated for normality using the Kolmogorov–Smirnov test, which revealed a normal distribution for all variables except urinary albumin excretion and triglyceride levels, which exhibited a positively skewed distribution. These non-normally distributed variables were reported as medians and interquartile ranges and were log-transformed prior to further statistical analysis. Normally distributed continuous variables were presented as means and standard deviations. Categorical variables were expressed as percentages.

Differences between groups were assessed using the independent *t*-test for continuous variables and the chi-squared test or, when appropriate, Fisher’s exact test for categorical variables. Potential confounders were adjusted using the analysis of covariance (ANCOVA).

To examine the relationship between retinal vascular densities and the other variables, simple linear regression analysis and Pearson’s correlation coefficients were employed. To assess the independent contribution of retinal vascular densities, multiple linear regression models were built, with each retinal vascular density variable as the dependent variable and the parameters that demonstrated significant associations with both the renal resistive index (RRI) and the retinal vascular densities in univariate analyses as independent variables.

Additionally, stepwise logistic regression analysis was conducted, using RRI> or <the 75th percentile as the dependent variable, with the retinal vascular densities, the blood pressure values, and the other clinical parameters as explanatory variables.

The null hypothesis was rejected for all two-tailed tests with *p*-values < 0.05.

## 3. Results

The mean RRI in the entire study population was 0.616 ± 0.06.

Table 1 presents the main demographic, anthropometric, and clinical characteristics of both the entire study population and the two groups in which the patients were categorized based on them being above or below the 75th percentile of the distribution of RRI (0.66, 95% CI 0.64–0.68).

Table 2 illustrates the distribution of pharmacologically treated patients, including the various antihypertensive drugs and other medications targeting the cardiovascular system.

While the percentage of subjects treated for hypertension did not differ significantly between the two groups, a higher prevalence of patients receiving centrally acting anti-adrenergic agents was observed in the group with RRI > 75th percentile.

Among the hemodynamic parameters, 24 h pulse pressure and daytime and nighttime pulse pressure were significantly higher in the group with RRI > 75th percentile (Table 3).

Regarding the retinal vascular density parameters, the only significant difference between the two groups was observed at the level of the deep foveal vascular plexus, which showed lower density in the group with the higher RRI (Table 4).

This difference remained statistically significant even after correction through the analysis of covariance (ANCOVA) for eGFR, mean nighttime pulse pressure, triglyceridemia, and smoking (*p* = 0.01 and *p* < 0.001, respectively).

Table 5 presents the statistically significant correlations between RRI and the various parameters, including demographic factors, markers of renal damage, glomerular filtration rate, nighttime pulse pressure, and deep foveal vascular density.

**Figure 1:**
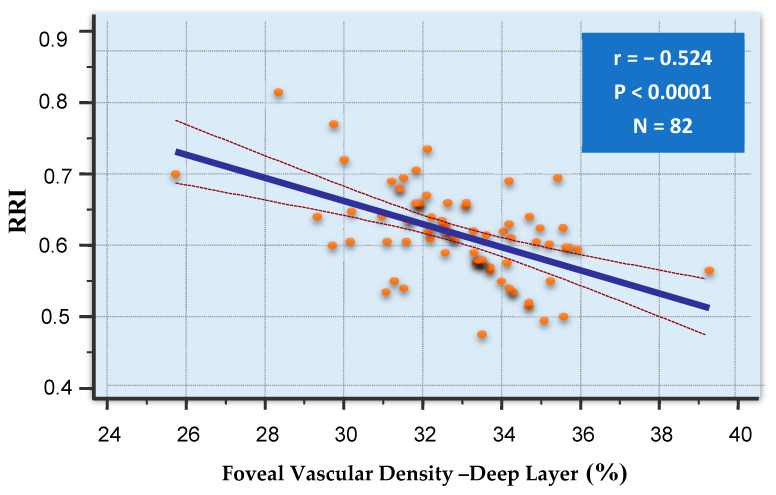
Correlation between RRI and Deep Foveal Vascular Density. (Blue Line represents the linear regression trendline, showing the negative correlation between the two variables. The slope indicates the strength and direction of the relationship; dotted lines represents the confidence intervals for the regression line; orange dots represents re individual data points.

The inverse relationship between the RRI and the deep foveal density appeared particularly strong (Figure 1).

In the group of patients in which the semiquantitative analysis of microalbuminuria was positive, comprising 18 subjects, a significant inverse correlation was observed between the logarithm of urinary albumin excretion and the superficial parafoveal vascular density (*r* = −0.555; *p* < 0.001). The small size of this subgroup does not allow us to perform statistical corrections to assess whether this relationship is independent of potential confounding factors.

The association between renal resistive index and deep foveal vascular density was tested in multivariate models where, alternately, RRI and deep foveal vascular density were considered as dependent variables (see Table 6 and Table 7). In both cases, the relationships between these two variables remained largely significant.

Moreover, in multiple logistic regression analysis, an increase in mean nighttime pulse pressure (OR = 1.1319, CI 1.049–1.221) and a reduction in deep foveal vascular density (OR = 0.5026) (Table 8) are independently associated with a higher likelihood of an RRI above the 75th percentile.

## 4. Discussion

It is widely recognized that damage to small vessels exerts a comparable effect on morbidity and mortality, particularly due to the impairment of cerebral and renal microcirculations, which are especially vulnerable to fluctuations in systemic pulsatile blood flow. In this context, albuminuria, although not universally associated with microvascular damage, is regarded as a biomarker of microvascular dysfunction and serves as an independent predictor of both morbidity and mortality [21].

The assessment of macrovascular and microvascular circulation is essential for the timely and accurate diagnosis of vascular abnormalities, playing a critical role in the primary and secondary prevention of cardiovascular diseases, as well as in determining the most effective therapeutic strategies, particularly in patients with hypertension or chronic kidney disease.

In the context of cardiovascular risk, an increased RRI, as an indicator of enhanced microvascular tone, is associated with the degree of renal impairment caused by elevated blood pressure. Several studies in the literature have reported significant correlations between RRI and various cardiovascular risk factors, such as left ventricular hypertrophy and carotid atherosclerosis [22,23,24]. Additionally, significant associations have been observed between RRI and aortic stiffness, measured both by pulse wave velocity and central pulse pressure [25].

The retina is a complex structure closely integrated with the cardiovascular system. It is one of the most metabolically active organs and consumes a significant amount of oxygen relative to its weight. A sophisticated network of blood vessels delivers oxygen and nutrients to the various layers of the retina. The inner two-thirds are supplied by capillary networks originating from the central retinal artery. The superficial vascular plexus is located within the ganglion cell layer, while the intermediate and deep vascular plexuses are positioned above and below the inner nuclear layer, collectively referred to as the deep capillary complex [26].

Vascular and/or cardiac dysfunction often impacts retinal health due to its intricate and complex vascular network.

Our study results demonstrate that ocular microvascular alterations, assessed via retinal capillary density analysis using OCTA, are clearly associated with a hemodynamic index obtained through Doppler ultrasound of renal arteries—the intraparenchymal renal resistive index. The RRI is a well-established prognostic marker for both renal disease progression and systemic macrovascular alterations.

Importantly, this association remains significant after adjusting for potential confounding factors such as glomerular filtration rate (eGFR), nighttime pulse pressure, triglycerides, smoking, and certain drugs. This relationship is biologically plausible due to the physiopathological connections between the kidney and the eye and aligns partially with our previous studies.

In one of these, which included patients with primary nephropathies, we observed a strong inverse relationship between renal function and retinal vascular density [27]. Similarly, findings from Chua et al. corroborate our results, showing an association between deep foveal vascular density and eGFR and office-based BP [28]. This suggests that systemic hypertension and arterial stiffness may impact one retinal vascular layer differently than the other and we can hypothesize that a sparser network of retinal capillaries, an expression of microvascular rarefaction, could reflect similar alterations in renal microcirculation.

However, these studies did not include an evaluation of the renal arteries, as our current study does.

Vascular density in the deep and superficial capillary plexuses has been found to be reduced in patients with hypertension, particularly in the macula in the other previous studies [29,30,31].

In patients diagnosed with hypertension, stratified by cardiovascular risk based on factors such as the presence of organ damage, blood glucose levels, and adiposity indices, an increased risk correlates with a lower perfusion density in both the superficial and deep capillary plexuses.

Our analysis did not reveal any significant associations with the density of the superficial foveal plexus.

In a meta-analysis of eleven studies, nine published results examined the superficial foveal plexus in 787 eyes of 659 patients with systemic hypertension and in 513 eyes of 449 healthy controls. The analysis revealed a significant reduction in vascular density in the eyes of patients with systemic hypertension compared to the control group ([SMD], −0.50 [−0.70, −0.30], *p* < 0.00001) [32].

Regarding the density of the deep foveal plexus, the same meta-analysis conducted on seven studies, which included a total of 618 eyes from 517 patients with systemic hypertension and 296 eyes from 232 healthy controls, demonstrated a significantly lower vascular density in patients with systemic hypertension compared to the healthy controls (SMD, −0.38 [−0.64, −0.13], *p* = 0.004).

In a subsequent study involving renal artery eco-Doppler analysis, we identified an inverse relationship between RRI and choroidal thickness [33].

Moreover, a recent Australian study of 150 hypertensive patients reported a connection between retinal capillary rarefaction, increased albumin-to-creatinine ratio, reduced eGFR, and elevated pulse wave velocity, with the latter being a known indicator of vascular stiffness [34]. Although our study did not measure pulse wave velocity, we observed that the nighttime pulse pressure, an indirect proxy for stiffness, was significantly and independently correlated with the RRI. This aligns with our previous research demonstrating a close relationship between the aortic pulse wave velocity and the intrarenal resistance indices [33,35].

In a previous study conducted by our research group to explore the relationship between parameters and renal dysfunction in hypertensive, non-diabetic patients, office-based blood pressure measurements were employed for data analysis. However, no significant correlations were observed between these variables. Therefore, we cannot dismiss the possibility that out-of-office blood pressure monitoring methods, such as 24 h ambulatory blood pressure monitoring or home-based blood pressure measurements, might reveal a link between ophthalmic variables and blood pressure parameters [36].

The strong relationship between systemic vascular stiffness and intrarenal resistive indices suggests that the observed association between RRI and retinal vascular density may be mediated by increased arterial stiffness. The pulsatile nature of central hemodynamics has a detrimental impact on vital organs. In a healthy cardiovascular system, the elasticity of large arteries buffers pulse and flow oscillations generated by cyclical left ventricular systoles, ensuring continuous microcirculatory flow. However, vascular aging, hypertension, and other cardiovascular risk factors reduce this buffering capacity, exposing the microcirculation to heightened pulsatile stress. This is particularly relevant for organs with high blood flow and low resistance, such as the brain, kidneys, and potentially the chorioretinal region [33].

These findings support the concept of “crosstalk” between the micro- and macrovasculature. Our results suggest that evaluating the retinal capillary network could provide indirect insights into the systemic arterial tree’s health. Additionally, the loss of statistical significance in the relationship between eGFR and retinal vascular density when RRI is included in the multivariate models suggests that previously observed associations between eGFR and the chorioretinal circulation are primarily mediated by vascular factors.

Nonetheless, given the relatively small sample size, number of data related to albuminuria and the cross-sectional design of this study, the results should be interpreted with caution. Further longitudinal and larger-scale studies are necessary to confirm these findings.

## 5. Conclusions

In conclusion, our results confirm the existence of a strong relationship between the renal and the ocular microcirculation and further support the hypothesis of intimate physiopathological connections between the micro- and macrocirculatory districts. Further prospective studies with a larger sample size are needed to confirm our findings.

## Figures and Tables

**Table 1:** Main demographic, anthropometric, clinical, and biochemical characteristics of the study population.

	Total (*n* = 92)	RRI < 75 pct	RRI > 75 pct	*p*
**Age, y**	48 ± 13	48 ± 12	52 ± 14	0.24
**Male sex, *n* (%)**	65 (79)	47 (78)	18 (83)	0.85
**BMI (kg/m^2^)**	28 ± 4.6	27.5 ± 4.6	29.4 ± 4.5	0.10
**Waist circumference (cm)**	96 ± 13	96 ± 13	99 ± 12	0.10
**Current smokers, *n* (%)**	20 (22.2)	11 (17.4)	9 (38.9)	0.054
**eGFR (mL/min/1.73 m^2^)**	87 ± 20	90 ± 19	76 ± 24	0.018
**Urinary albumin excretion (mg/day)**	76 (31–252)	36 (30–86)	387 (126–646)	0.009
**Hemoglobin (g/dL)**	14.6 ± 1.4	14.7 ± 1.3	14.2 ± 1.6	0.12
**Total Cholesterol (mg/dL)**	194 ± 29	196 ± 29	186 ± 26	0.14
**HDL-Cholesterol (mg/dL)**	48 ± 12	49 ± 12	45 ± 9	0.11
**Tryglicerides (mg/dL)**	118 (75–154)	106 (89–131)	122 (80–165)	0.02
**Fasting Blood glucose (mg/dL)**	96 ± 17	96 ± 18	97 ± 14	0.80

**Table 2:** Percentage of Pharmacologically Treated Patients.

	Total	RRI < 75 pct	RRI > 75 pct	*p*
**Pharmacologically treated hypertensive subjects**	67.1	69.4	58.8	0.60
**Antihypertensive drugs**				
Angiotensin-converting enzyme (ACE) inhibitors, %	29.1	27.4	35.3	0.57
Sartans, %	37.9	40.3	29.4	0.46
Calcium channel blockers, %	35.4	38.7	23.5	0.28
Alpha-2 Adrenergic Receptor Agonists, %	7.6	3.2	23.5	0.005
A-Blockers, %	27.8	29	23.6	0.57
α β-blockers, %	12.7	12.9	11.8	0.58
β-blockers, %	12.7	14.5	5.8	0.63
Diuretics %	35.4	33.9	41.2	0.71
**Other cardiovascular agents**				
Statins, %	11.4	9.7	17.6	0.71
Antiplatelet agents, %	25.3	29	11.8	0.57
Allopurinol, %	6.3	4.8	11.8	0.83

**Table 3:** Hemodynamic parameters of whole population and two subgroups.

	Total	RRI < 75 pct	RRI > 75 pct	*p*
**Office-based SBP (mmHg)**	137 ± 12	137 ± 13	136 ± 11	0.94
**Office-based DBP (mmHg)**	86 ± 9	87 ± 9	85 ± 9	0.83
**Office-based PP (mmHg)**	51 ± 9	50 ± 9	52 ± 12	0.83
**Heart Rate (bpm)**	73 ± 11	74 ± 12	72 ± 11	0.87
**Mean 24-h SBP (mmHg)**	130 ± 13	133 ± 13	138 ± 13	0.71
**Mean 24-h DBP (mmHg)**	82 ± 9	83 ± 9	82 ± 9	0.92
**24-h PP (mmHg)**	47 ± 10	45 ± 10	52 ± 9	0.013
**Daytime SBP (mmHg)**	134 ± 13	129 ± 13	134 ± 13	0.70
**Daytime DBP (mmHg)**	85 ± 9	86 ± 10	85 ± 9	0.92
**Daytime PP (mmHg)**	48 ± 11	47 ± 11	54 ± 9	0.038
**Nighttime SBP (mmHg)**	119 ± 14	117 ± 13	126 ± 15	0.03
**Nighttime DBP (mmHg)**	75 ± 10	75 ± 10	75 ± 10	1
**Nighttime PP (mmHg)**	44 ± 10	43 ± 9	51 ± 10	0.001

Abbreviations—SBP: systolic blood pressure; DBP: diastolic blood pressure; PP: pulse pressure.

**Table 4:** Retinal vascular density parameters of whole population and two subgroups.

	Total	RRI < 75 pct	RRI > 75 pct	*p*	*p* *
**Parafoveal Vascular Plexus Density (%)—Superficial Layer**	37.3 ± 0.87	37.4 ± 0.84	36.9 ± 0.89	0.053	0.052
**Parafoveal Vascular Plexus Density (%)—Deep Layer**	38.3 ± 1.08	38.3 ± 1.06	38.3 ± 1.19	0.956	0.876
**Foveal Vascular Density (%) ** **–Superficial Layer**	34.5 ± 1.88	34.7 ± 1.69	33.7 ± 2.34	0.147	0.110
**Foveal Vascular Density (%)** **–Deep Layer**	32.9 ± 1.95	33.26 ± 1.72	31.47 ± 2.11	0.01	0.001

*p* * after ANCOVA correction for eGFR, log-transformed triglycerides, mean nocturnal pulse pressure, and smoking).

**Table 5:** Correlations between RRI and other parameters.

		Age	eGFR	Nighttime PP	UAE	Deep Foveal Plexus Vascular Density
**(RRI)**	r=	0.235	−0.288	0.3027	0.555 *	−0.524
	*p*=	0.0336	0.0087	0.0057	<0.001	<0.001

* The data related to UAE* refer to the patients who tested positive in the semi-quantitative evaluation of this parameter and subsequently underwent quantification through a 24-h urine collection.

**Table 6:** Multiple linear regression analysis.

Dependent Variable: RRI	B *	SE	R Partial	*p*
Foveal Vascular Density (%) –Deep Layer	−0.0158	0.0029	−0.549	<0.0001
Mean nighttime PP (mmHg)	0.0020	0.0006	0.3063	0.0007
Constant	1.0468			

* B unstandardized regression coefficient. SE standard error; R correlation coefficient. Other variables that did not reach statistical significance include eGFR, LogT, smoking, centrally acting anti-adrenergic drugs.

**Table 7:** Multiple linear regression analysis.

Dependent Variable: Foveal Vascular Density (%) –Deep Layer	B *	SE	R Partial	*p*
RRI	−18.98	3.43	−0.549	<0.0001
Mean nighttime PP (mmHg)	0.048	0.0027	0.2615	0.025
Constant	42.38			

* B unstandardized regression coefficient. SE standard error; R correlation coefficient. Other variables that did not reach statistical significance include eGFR, LogT, smoking, centrally acting anti-adrenergic drugs.

**Table 8:** Multiple logistic regression analysis.

Dependent Variable: RRI> or <75 pct	Odds Ratio	95% CI	*p*
Covariates			
**Foveal Vascular Density (%)** **–Deep** **Layer**	−0.5026	0.3129–0.8073	0.0044
**Mean nighttime PP (mmHg)**	1.1319	1.0493–1.2211	0.0014
**Constant**	15.3587		

Other variables that did not reach statistical significance include eGFR, LogT, smoking, centrally acting anti-adrenergic drugs.

## Data Availability

The datasets generated during and/or analyzed during the current study are available from the corresponding author on reasonable request.
